# Minimum Dietary Diversity and Associated Factors Among 6–23 Months Children in Ethiopia: A Systematic Review and Meta‐Analysis

**DOI:** 10.1002/fsn3.72481

**Published:** 2026-10-13

**Authors:** Melkamu Senbeta Jimma, Dawit Getachew Assefa, Muluwas Amentie Zelka, Alemayehu Assefa Amaya, Mulualem Birhanu Begawo, Ermias Sisay Chanie, Fisha Alebel Gebreeyesus, David Lim

**Affiliations:** ^1^ Center for Improving Palliative, Aged and Chronic Care Through Clinical Research and Translation University of Technology Sydney Ultimo New South Wales Australia; ^2^ Department of Nursing, College of Health Science Assosa University Assosa Ethiopia; ^3^ Department of Nursing, College of Health Science and Medicine Dilla University Dilla Ethiopia; ^4^ Department of Public Health, College of Health Science Assosa University Assosa Ethiopia; ^5^ Department of Pediatrics and Child Health Nursing, College of Health Science Debre Tabor University Debre Tabor Ethiopia; ^6^ University of New South Wales Sydney Australia; ^7^ Department of Nursing, College of Health Science Wolkite University Wolkite Ethiopia

**Keywords:** age 6–23 months, dietary diversity, Ethiopia, prevalence, systematic review

## Abstract

The 6–23‐month period is a critical stage in child development, during which the transition from exclusive breastfeeding to complementary feeding increases the risk of nutritional deficiencies and related health problems. This study aimed to estimate the prevalence of minimum dietary diversity (MDD) and identify factors associated with MDD among children aged 6–23 months in Ethiopia. A systematic review and meta‐analysis were conducted in accordance with PRISMA guidelines. PubMed, Google Scholar, Embase, Health InterNetwork Access to Research Initiative (HINARI), Cochrane Library, and Web of Science were searched for studies published from April 1, 2018, to April 25, 2026. Study quality was assessed using the Newcastle‐Ottawa Scale. Thirteen studies were included and analyzed in RStudio version 4. A random‐effects model was used to estimate the pooled prevalence of MDD. Factors associated with MDD were synthesized narratively because of variable categorizations, directions of association, reference categories, and reporting of effect measures across studies. Subgroup analyses and an assessment of publication bias were also conducted. The review protocol was registered in PROSPERO (CRD42023415050). Thirteen studies involving 7194 participants were included. The pooled prevalence of minimum dietary diversity among Ethiopian children aged 6–23 months was 27% (95% CI: 21–33). Substantial between‐study heterogeneity was observed (*I* ^2^ = 97.58%, *p* < 0.001; *τ* ^2^ = 0.01; Q(12) = 414.67, *p* < 0.001), indicating considerable variability in prevalence estimates across studies and warranting cautious interpretation. Among 13 studies that adjusted for confounders, child age, maternal education, use of antenatal and postnatal care, family size, and household wealth index were the most frequently reported as being significantly associated with MDD in the primary studies. In conclusion, despite observed heterogeneity, the prevalence of MDD remains low and below the WHO 2030 target. Strengthening maternal education, antenatal and postnatal care, and socioeconomic conditions may improve child nutrition and support progress toward Sustainable Development Goal 2.2 by 2030.

## Graphical abstract

A systematic review and meta‐analysis of 13 studies involving 7194 children aged 6–23 months in Ethiopia found a pooled prevalence of minimum dietary diversity (MDD) of 27% (95% CI: 21%–33%). The most frequently reported factors associated with MDD in primary studies were child age, maternal education, antenatal care, postnatal care, household wealth index, and family size. Strengthening maternal education, antenatal and postnatal care, and socioeconomic conditions may improve dietary diversity and support progress toward Sustainable Development Goal (SDG) 2.2 by 2030.

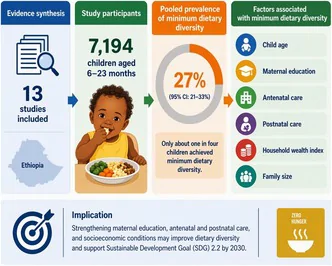

## 1 Introduction

Inadequate dietary diversity negatively affects children's health and may contribute to increased economic burdens on both households and the government (Hasanah et al. 2024). Low dietary diversity has been significantly associated with linear growth faltering and developmental delays in children (George et al. 2022), with consequences that can persist across generations.

Malnutrition during the first 1000 days of life is strongly linked to long‐term adverse health and well‐being outcomes (Wells et al. 2019). Limited dietary diversity negatively impacts physical capacity, cognitive development, and social well‐being (Ray and Dutta 2022). Dietary diversity, defined as the number of food groups consumed by an individual or household over time, is considered a proxy for adequate intake of essential nutrients (Ray and Dutta 2022).

Proper feeding practices for infants and young children during the first 2 years of life are critical for optimal growth, health, and development (Beyene et al. 2015). Complementary feeding, the introduction of solid or semi‐solid foods alongside breast milk, is essential for meeting children's increasing nutritional demands (Beyene et al. 2015). According to the World Health Organization (WHO), children aged 6 months to 2 years should receive appropriate complementary feeding that includes appropriate food texture, minimum meal frequency, and minimum dietary diversity (MDD) (Bhujel et al. 2021).

For children aged 6–23 months, the MDD score is a population‐level indicator developed by the WHO to assess dietary diversity (Tufts University 2023). It is one of eight Infant and Young Child Feeding (IYCF) indicators designed to provide simple, valid, and reliable metrics for evaluating feeding practices (World Health Organization 2008). The proportion of children meeting the MDD criteria is also a core process indicator in the Global Nutrition Monitoring Framework (World Health Organization 2023).

Inadequate dietary diversity is directly linked to poor growth outcomes, including wasting, underweight, and their severe forms (Saha et al. 2022). In contrast, higher dietary diversity is associated with better health outcomes and a reduced risk of chronic diseases, while lower diversity negatively impacts physical capacity, cognitive development, and social well‐being (Ray and Dutta 2022).

Although dietary diversity is strongly associated with stunting, some studies suggest that this association may be consistent across different household economic levels (Paramashanti et al. 2017). An individual's dietary diversity score is widely recognized as a useful indicator of overall dietary quality (Ray and Dutta 2022; Paramashanti et al. 2017; Rah et al. 2010; Trisasmita et al. 2020).

However, fewer than one‐third of children aged 6–23 months in Africa meet the MDD criteria (Beyene et al. 2015), highlighting a significant gap in child nutrition across the continent.

A recent review of stunting risk factors in sub‐Saharan Africa found that the high prevalence of stunting is closely linked to inadequate child‐feeding practices (Quamme and Iversen 2022). Poor complementary feeding significantly increases the risk of undernutrition, illness, and mortality among children under age two (World Health Organization 2009). Evidence from India's National Family and Health Survey also shows that insufficient dietary diversity is associated with severe stunting, wasting, and underweight in children (Saha et al. 2022). More than two‐thirds of malnutrition‐related child deaths are attributed to inappropriate feeding practices during the first 2 years of life (Black et al. 2008). Poor maternal, infant, and young child feeding practices have been strongly linked to high levels of malnutrition (Fanzo et al. 2018). Children who survive malnutrition often face long‐term health consequences and increased morbidity, with effects that may extend across generations (World Health Organization 2009).

Childhood undernutrition remains a major public health concern in resource‐limited settings. Despite global progress, approximately 22.2% of children under five are stunted, 50.5 million suffer from wasting, and 16 million experience both conditions simultaneously (United Nations Children's Fund (UNICEF) 2019). In Ethiopia, despite efforts to improve dietary diversity, the problem persists and varies across regions.

Despite this growing body of research, a comprehensive, up‐to‐date systematic review and meta‐analysis are needed to consolidate the evidence and guide the development of effective interventions. The relationship between dietary diversity and its associated factors remains underexplored. This study aims to synthesize the pooled prevalence of MDD among children aged 6–23 months in Ethiopia and identify its key determinants. The findings are intended to inform national policy, improve child nutrition practices, and support progress toward Sustainable Development Goal 2.2.

### 1.1 Research Question

What is the prevalence of MDD and its associated factors among children aged 6–23 months in Ethiopia?

## 2 Methods and Materials

### 2.1 Research Design and Methods

The Preferred Reporting Items for Systematic Review and Meta‐Analysis (PRISMA) guidelines for observational studies were used to prepare the systematic review (Page et al. 2012). The revised and accessible protocol was registered with PROSPERO (CRD42023415050).

### 2.2 Information Sources

The following database searches were updated on April 25, 2026: PubMed (NLM), Embase (Ovid), Web of Science Core Collection (Clarivate), the Health InterNetwork Access to Research Initiative (HINARI), the Cochrane Library, and Google Scholar.

### 2.3 Inclusion and Exclusion Criteria

Studies were included if they reported the prevalence of MDD feeding practices, were conducted in Ethiopia, involved children aged 6–23 months, were published in peer‐reviewed journals since 2018, used a community‐based cross‐sectional design, were articles or abstracts published in English, and reported factors associated with MDD.

Studies were excluded if they did not report the outcome of interest, were not relevant to the study context, included interventions outside the scope of this review, used non‐cross‐sectional study designs, involved populations that did not meet the eligibility criteria, were inaccessible, were conducted in special, emergency, or conflict‐affected areas, were not published in English, or did not report scientific outcomes related to MDD practice.

### 2.4 Search Strategies

A comprehensive literature search was conducted from database inception to 25 April 2026 in PubMed (NLM), Embase (Ovid), Web of Science Core Collection (Clarivate), HINARI, the Cochrane Library, and Google Scholar. The search strategy was developed using key concepts related to MDD, complementary feeding, associated factors/determinants, children aged 6–23 months, and Ethiopia. Controlled vocabulary terms, including medical subject headings (MeSH) in PubMed and Emtree terms in Embase, were combined with free‐text keywords using the Boolean operators “AND” and “OR.” Searches were limited to studies published in English. No publication date restrictions were applied. Reference lists of all eligible studies were also screened manually to identify additional relevant articles. The complete database‐specific search syntaxes used in this review are attached in File S1.

### 2.5 Data Extraction

Data extraction was conducted using a standardized Microsoft Excel form, following a pilot test on a random sample of ten studies. The extraction sheet captured key information, including first author, year, region, study year, publication year, study design, number of cases, total sample size, sample size used, prevalence of MDD, and NOS quality score. Additionally, data on associated factors and their corresponding effect estimates (AORs and 95% CIs) were extracted when available.

Two investigators (MSJ and DGA) independently extracted the data, and an audit was conducted to ensure accuracy and consistency. Any disagreements arising during study selection, quality assessment, or data extraction were resolved through discussion and consensus. Where disagreements persisted, a final decision was reached following consultation with the research team (MAZ, MBB, AAA). Since no data were missing, the authors were not contacted. Only verified data were included in the final analysis.

### 2.6 Risk of Bias Assessment in Included Studies

The quality of the included studies was assessed using the Newcastle‐Ottawa Scale (Carra et al. 2025). No studies were excluded based on quality assessment, as all were found to be at low risk of bias. This assessment was conducted independently by MSJ and FAG and cross‐checked by DL and DGA.

### 2.7 Variables

#### 2.7.1 Outcome Variable

MDD was assessed using both the 2008 and 2021 WHO indicators because the studies included in this review used either definition. To ensure consistency with the original studies and avoid misclassification of the outcome measures, subgroup analyses were conducted separately for each MDD definition used in the original studies. This approach enabled comparison of findings across studies that used different WHO recommendations. MDD is defined as the proportion of children aged 6–23 months who consumed at least 4 of 7 food groups in the previous 24 h. The food groups are: (1) grains, roots, and tubers; (2) legumes and nuts; (3) dairy products; (4) flesh foods (meat, fish, poultry, liver/organ meats); (5) eggs; (6) provitamin A‐rich fruits and vegetables; and (7) other fruits and vegetables (World Health Organization 2008).

MDD is defined as the proportion of children aged 6–23 months who consumed ≥ 5 of 8 food groups in the previous 24 h. The food groups include the 7 listed above and an additional breastmilk group (World Health Organization 2017).

#### 2.7.2 Independent Variables

Child‐related, maternal, household, and socioeconomic factors. Child‐related factors included age (e.g., 6–11, 12–17, and 18–23 months). Maternal factors included educational status (no formal education, primary, secondary, or higher education), utilization of antenatal (yes/no), postnatal care (yes/no), and knowledge of dietary diversification. Household and socioeconomic factors included household wealth index (e.g., poor, middle, and rich), family size, and father's occupation (e.g., farmer, merchant, employee, and other occupations). These variables were assessed across the included studies using different categories to determine their association with MDD among children aged 6–23 months.

### 2.8 Synthesis and Analysis of Data

Heterogeneity was present across studies, as indicated by an *I* ^2^ value greater than 75%; therefore, a random‐effects model was employed to estimate the prevalence of MDD (Borenstein 2023). For consistency of reporting, *I* ^2^ values were reported together with *τ* ^2^ and the Q statistic, along with corresponding *p*‐values. Pooled proportions were reported to assess the prevalence of MDD among children aged 6–23 months in Ethiopia.

Subgroup analyses by year of publication, sample size, region, and MDD definition were conducted to assess heterogeneity. Forest plots were generated to visualize the pooled estimates, and funnel plots were used to assess potential publication bias or small‐study effects. R software was used for all statistical analyses, consistent with the conventional approach (Balduzzi et al. 2019).

A meta‐analysis of factors associated with MDD was not performed because of substantial variation in variables, outcome definitions, the direction of associations, categories and classifications, and the reporting of effect measures across the included studies. Therefore, factors associated with MDD were synthesized narratively. Variables reported as statistically significant in the original studies were extracted, grouped by conceptual similarity, and summarized descriptively. The narrative synthesis identified the most frequently reported factors associated with MDD, based on the number and proportion of included studies that reported statistically significant associations. No pooled effect estimates were calculated for these factors.

## 3 Result

### 3.1 Search Results and Selection Process

Search results from the databases were imported into EndNote X7.3 (Clarivate, PA) for screening and reference management. Duplicate records were identified and removed before screening. Two reviewers (MSJ and DGA) independently screened the titles and abstracts of all retrieved records against the predefined inclusion criteria. Studies were screened based on the outcome of interest, population, study design, geographical setting, and publication eligibility criteria. Each study identified in the initial screening was independently assessed by two reviewers (MSJ and DGA) using a standardized form aligned with the predefined inclusion criteria.

Full‐text articles of potentially eligible studies were then retrieved and independently assessed for eligibility by the same reviewers. Any disagreements about study eligibility were resolved through discussion and consensus. When consensus could not be reached, a third reviewer was consulted to make the final decision.

As shown in Figure 1, 997 records were retrieved from the database searches. After removing 99 duplicate entries, 898 records underwent title and abstract screening. Of these, 857 were excluded as irrelevant. The full texts of the remaining 41 articles were assessed for eligibility, and 28 studies were excluded for incorrect setting, outcome, intervention, study design, or population; 13 studies were included in the final review.

**FIGURE 1:**
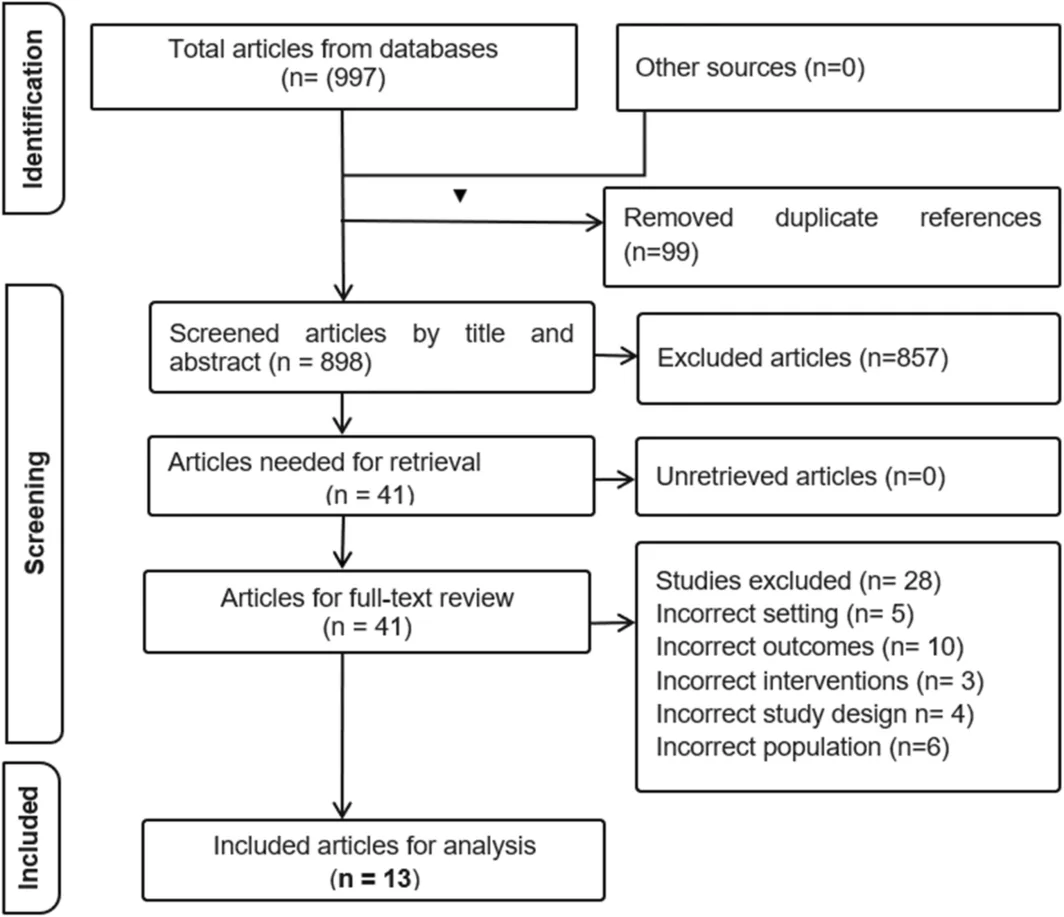
PRISMA flow diagram of the prevalence of minimal dietary diversity among children aged 6–23 months in Ethiopia (2018 to 2026).

### 3.2 Characteristics of Included Studies

The included studies were published between April 1, 2018, and April 25, 2026. Five studies were conducted in the Southern Nations, Nationalities, and People's Region (SNNPR) of Ethiopia (Dafursa and Gebremedhin 2019; Darebo et al. 2025; Gezahegn and Tegegne 2020; Habtamu et al. 2021; Molla et al. 2021), three in the Oromia region (Bedada Damtie et al. 2020; Belete et al. 2022; Keno et al. 2021), three in the Amhara region (Assefa and Belachew 2022; Mulatu, Ejigu, et al. 2024; Yesuf et al. 2021), and one each in Dire Dawa (Sema et al. 2021) and Afar (Wuneh et al. 2019). Three studies were conducted at the zonal level (Molla et al. 2021; Belete et al. 2022; Mulatu, Ejigu, et al. 2024), five at the district level (Dafursa and Gebremedhin 2019; Darebo et al. 2025; Belete et al. 2022; Keno et al. 2021; Assefa and Belachew 2022), four at the town level (Dafursa and Gebremedhin 2019; Habtamu et al. 2021; Bedada Damtie et al. 2020; Yesuf et al. 2021), and one at the city level (Sema et al. 2021) (see Table 1). Sample sizes ranged from 335 to 807, for a total of 7194 participants. Response rates across studies ranged from 96% to 100%.

**TABLE 1:** Characteristics of studies included in the systematic review and meta‐analysis of MDD among children aged 6–23 months in Ethiopia (2018–2026).

First author, year	Region	Study year	Publication year	Study design	Number of cases	Total sample size	Sample size used	Prevalence of MDD, % (95% CI)	Associated factors effect estimate (95% CI)	NCO quality score
Sema et al. 2021	Dire Dawa	2019	2021	Cross‐sectional	107	451	438	24.40 (95% CI: 20.3, 28.5)	Maternal education (AOR 2.20; 95% CI: 1.08, 4.52), decision‐making (AOR = 2.5; 95% CI: 1.19, 5.29), antenatal care (AOR = 2.19; 95% CI: 1.20, 3.99), postnatal care (*p* < 0.011, AOR = 6.4; 95% CI: 2.78, 14.94) and facility delivery (AOR = 2.66; 95% CI: 1.35, 5.25) were maternal factors. Moreover, child's age (*p* < 0.04, AOR = 2.84; 95% CI: 1.39, 5.83), and child's sex (AOR = 2.85; 95% CI: 1.64, 4.94)	8
Molla et al. 2021	SNNPR	2019	2021	Cross‐sectional	199	691	665	29.90	Age of children (*p* < 0.00, AOR 4.237) (1.743–10.295), Educational status (*p* < 0.00, AOR 2.864) (1.156–7.094), Number of families (AOR 2.865) (*p* < 0.00, 1.776–4.619) and household wealth index (*p* < 0.00, AOR 4.390) (2.300–8.380)	8
Belete et al. 2022	Oromia	2019	2022	Comparative cross‐sectional	176	686	674	26.10 (95% CI 22.8, 29.5)	Occupation (merchant mother), own production of foods at the household level, frequent advice of IYCF practices during postnatal care (PNC) visit in urban residents, maternal secondary educational level, living with caregiver only, having a merchant father, advice of IYCF practice during PNC visit, and utilization of horse as a means of transportation in rural areas	7
Yesuf et al. 2021	Amhara	2018	2021	Cross‐sectional	228	517	511	44.60 (95% CI: 40.1%–49%)	Child age (6–11 months) (*p* < 0.05, AOR = 0.55 (0.34, 0.89)), father's occupation (*p* < 0.05, AOR = 2.41 ((95% CI: 1.16, 5.01)), father's educational status (primary and below) (AOR = 0.33 (95% CI: 0.14, 0.76)), housewife mothers/caregivers OR = 2.41 (AOR 95%: 1.16, 5.01)), did not have child history of illness in the last 1 week AOR = 1.58 (95% CI: 1.01, 2.48), and mothers' knowledge on dietary diversity (*p* < 0.01, AOR = 0.35 (95% CI: 0.23, 0.55))	8
Wuneh et al. 2019	Afar	2018	2019	Cross‐sectional	175	807	804	21.80 (95% CI: 40.4%–47.2%)	Maternal education (AOR = 2.5, 95% CI = 1.1–5.3 and AOR = 3.9, 95% CI = 1.3–11.5), maternal occupation (AOR = 4.2, 95% CI = 2.3–7.8), sex of child (AOR = 2.6, 95% CI = 1.5–4.5) and history of postnatal care visit (AOR = 1.8, 95% CI = 1.1–3.2), age of child (AOR = 2.8, 95% CI = 1.4–5.5 and AOR = 5.3, 95% CI = 2.3–12.4), sex of child (AOR = 2.6, 95% CI = 1.4–4.6) and history of postnatal care visit (AOR = 2.2, 95% CI = 1.3–3.8)	8
Keno et al. 2021	Oromia	2020	2021	Cross‐sectional	110	631	631	17.32 (95% CI (0.14–0.20))	Children aged (18–23) months (*p* < 0.05, AOR = 3.26, 1.36–7.79), mothers aged (35–44) years (AOR = 3.25, 1.38–7.45), housewives as household heads (AOR = 3.41, 1.56–2.37), children of smaller family size (*p* < 0.05, AOR = 3.89, 1.18–12.78), and caregivers who studied grade 9–12 (AOR = 9.98, 5.66–17.10), who received information about food diversity during ANC (*p* < 0.05, AOR = 1.48, 2.21–11.23) and PNC (*p* < 0.05, AOR = 3.94, 2.04–7.63) visit, who travels less than 1 h to reach the market (AOR = 2.94, 1.24–6.91) and who had high family income (*p* < 0.05, AOR = 4.12, 1.90–8.19)	8
Assefa and Belachew 2022	Amhara	2020	2022	Cross‐sectional	94	530	512	18.20 (CI: 14.8, 21.7)	Availability of cow's milk at household (AOR = 17.27; 95% CI: 6.73, 44.44), cultivating vegetables (AOR = 3.2; 95% CI: 1.05, 9.8), availability of farmland (AOR = 10.15, 95% CI: 1.78, 57.93), and number of animals (AOR = 6.46; 95% CI: 1.97, 21.12)	8
Gezahegn and Tegegne 2020	SNNPR	2018	2023	Cross‐sectional	200	517	503	39.80 (95% CI 35.52, 44.08)	Postnatal care visit (*p* < 0.05, AOR =1.9, 95% CI 1.3, 2.8) and attending growth monitoring follow‐up (AOR = 1.5, 95% CI 1.001, 2.2)	7
Bedada Damtie et al. 2020	Oromia	2017	2020	Cross‐sectional	118	517	508	23.00	Children aged (12–23) months (*p* < 0.05, AOR = 2.99) (1.65, 5.42), maternal education (secondary and above) (*p* < 0.05, AOR = 3.21) (1.05, 9.85), had media exposure (*p* < 0.05, AOR = 3.91 (1.97, 7.77), maternal knowledge of diet diversification *p* < 0.05, AOR 8.50, 4.95, 14.58), and father's occupation (merchants) *p* < 0.05, AOR 2.27 (1.19, 4.33)	7
Dafursa and Gebremedhin 2019	SNNPR	2016	2019	Cross‐sectional	61	509	502	12.00 (95% CI: 9.0%–15.0%)	Maternal knowledge, *β =* 0.21 (*p* = 0.004), household food insecurity = 0.13 reduction in DDS (*p* = 0.001), household wealth index (*β* = 0.54, *p* = 0.041), father's literacy (*β* = 0.48, *p* = 0.002), ownership of home garden (*β* = 0.38, *p* = 0.01), mother's participation in cooking demonstrations (*β* = 0.19, *p* = 0.036), and child age in months (*β* = 0.04, *p* = 0.001), IYCF information via mass media (*β* = 0.04, *p* = 0.001) and during antenatal (*β* = 0.91, *p* = 0.022) and postnatal checkups (*β* = 0.21, *p* = 0.043)	7
Habtamu et al. 2021	SNNPR	2018	2021	Cross‐sectional	42	335	334	12.60 (95% CI; 9.2, 16.6)	A household with backyard gardening 2.34 (1.11, 5.10), maternal education (primary) *p* < 0.05, AOR = 0.30 (0.12, 0.81)	7
Darebo et al. 2025	SNNPR	2023	2025	Cross‐sectional	212	597	597	35.5 [95% CI 31.5, 39.7]	Equal decision‐making power (AOR: 2.46, 95% CI (1, 24, 4.9)), participation in cooking demonstrations (AOR = 1.61, 95% CI (1.10, 2.363)), attendance at ANC follow‐ups (AOR = 5.027, 95% CI (2.02, 12.46)), have nutrition counseling (AOR = 2.450, 95% CI (1.32, 4.54)), food secured family (AOR = 2.7, 95% CI (1.8, 4.06)), and child growth and development‐monitoring behavior (AOR = 1.8, 95% CI 1.24, 2.6)	8
Mulatu, Ejigu, et al. 2024	Amhara	2023	2024	Cross‐sectional	192	406	403	47.6	Maternal education (AOR 2.36, 95% CI (1.297, 3.957)), birth interval (AOR 2.85, 95% CI (1.45, 4.25)), and food insecurity (AOR = 2.23, 95% CI (1.626, 3.1))	8
						7194				

Abbreviations: MDD, minimum dietary diversity; NCO, newcastle ottawa scale.

### 3.3 Synthesis of Factors Affecting MDD

A meta‐analysis of factors associated with MDD was not conducted because variable categorizations were inconsistent, associations varied in direction, reference categories differed, and the number of studies reporting each factor was inadequate. Therefore, factors associated with MDD were synthesized narratively.

Among the 13 studies that adjusted for potential confounders, child age was the most frequently reported factor associated with MDD, being identified as statistically significant in 7 studies (53.8% of the included studies) (Dafursa and Gebremedhin 2019; Molla et al. 2021; Bedada Damtie et al. 2020; Keno et al. 2021; Yesuf et al. 2021; Sema et al. 2021; Wuneh et al. 2019). Maternal education was reported as significantly associated with MDD in 6 studies (46.1%) (Habtamu et al. 2021; Molla et al. 2021; Bedada Damtie et al. 2020; Belete et al. 2022; Mulatu, Ejigu, et al. 2024; Sema et al. 2021). Antenatal care utilization (Darebo et al. 2025; Keno et al. 2021; Sema et al. 2021), postnatal care utilization (Gezahegn and Tegegne 2020; Sema et al. 2021; Wuneh et al. 2019), maternal knowledge of food diversification (Bedada Damtie et al. 2020; Keno et al. 2021; Yesuf et al. 2021), household wealth index (Dafursa and Gebremedhin 2019; Molla et al. 2021; Keno et al. 2021), and the father's occupation (Bedada Damtie et al. 2020; Belete et al. 2022; Yesuf et al. 2021) were each reported as significantly associated factors in 3 studies (23.0%). Family size was reported as a significantly associated factor in 2 studies (15.4%) (Molla et al. 2021; Keno et al. 2021) (Table 2).

**TABLE 2:** Factors reported as statistically significant associated with MDD in the included studies among children aged 6–23 months in Ethiopia (2018–2026).

Statistically associated factors	Studies reported significant associations in *n* (%)	Effect estimate (AOR/*β*, 95% CI), and *p* reported in individual studies (References)
Child age	7 (53.8%)	AOR = 4.24 (95% CI: 1.743–10.295), *p* < 0.01 (Molla et al. 2021), AOR = 2.99 (95% CI: 1.65–5.42), *p* < 0.005 (Bedada Damtie et al. 2020), AOR = 3.26 (95% CI: 1.36–7.79), *p* < 0.005 (Keno et al. 2021), AOR = 0.55 (95% CI: 0.34–0.89), *p* < 0.005 (Yesuf et al. 2021), *β* = 0.04, *p* < 0.01 (Dafursa and Gebremedhin 2019), AOR = 2.84 (95% CI: 1.39–5.83), *p* < 0.04 (Sema et al. 2021), AOR = 5.3 (95% CI: 2.3–12.4), *p* < 0.005 (Wuneh et al. 2019)
Maternal education	6 (46.1%)	AOR = 0.30 (95% CI: 0.12–0.81), *p* < 0.05 (Habtamu et al. 2021), AOR = 2.86 (95% CI: 1.156–7.094), *p* < 0.01 (Molla et al. 2021), AOR = 3.21 (95% CI: 1.05–9.85) *p* < 0.005 (Bedada Damtie et al. 2020), *β* = 0.80 (95% CI: 0.41–1.19), *p* < 0.001 (Belete et al. 2022), AOR = 2.59 (95% CI: 1.26–3.32), *p* < 0.05 (Mulatu, Ejigu, et al. 2024), AOR = 2.20 (95% CI: 1.08–4.52), *p* < 0.031 (Sema et al. 2021)
ANC	3 (23.0%)	AOR = 6.38 (95% CI: 2.6–15.4), *p* < 0.001 (Darebo et al. 2025), AOR = 2.48 (95% CI: 2.21–11.23), *p* < 0.005 (Keno et al. 2021), AOR = 2.19 (95% CI: 1.20–3.99), *p* < 0.011 (Sema et al. 2021)
PNC	3 (23.0%)	AOR = 1.9 (95% CI: 1.3–2.8), *p* < 0.05 (Gezahegn and Tegegne 2020) AOR = 6.4 (95% CI: 2.78–14.94), *p* < 0.01 (Sema et al. 2021), AOR = 1.8 (95% CI: 1.1–3.2), *p* < 0.005 (Wuneh et al. 2019)
Maternal knowledge of food diversification	3 (23.0%)	AOR = 8.50 (95% CI: 4.95–14.58), *p* < 0.005 (Bedada Damtie et al. 2020), AOR = 1.48 (95% CI: 2.21–11.23), *p* < 0.005 (Keno et al. 2021), AOR = 0.35 (95% CI: 0.23–0.55), *p* < 0.001 (Yesuf et al. 2021)
Household wealth index	3 (23.0%)	*β* = 0.54, *p* < 0.041 (Dafursa and Gebremedhin 2019), AOR = 10.43 (95% CI: 5.288–20.57), *p* < 0.001 (Molla et al. 2021), AOR = 4.12 (95% CI: 1.90–8.19), *p* < 0.005 (Keno et al. 2021)
Father's occupation	3 (23.0%)	AOR = 2.27 (95% CI: 1.19–4.33), *p* < 0.005 (Bedada Damtie et al. 2020), *β* = 0.52 (95% CI: 0.10–0.94), *p* < 0.016 (Belete et al. 2022), AOR = 1.68 (95% CI: 1.00–2.84), *p* < 0.005 (Yesuf et al. 2021)
Family size	2 (15.4%)	AOR = 2.86 (95% CI: 1.776–4.619), *p* < 0.01 (Molla et al. 2021), AOR = 5.58 (95% CI: 1.73–17.91), *p* < 0.005 (Keno et al. 2021)

Abbreviations: β, regression coefficient; AOR, adjusted odds ratio; CI, confidence interval.

The highest reported prevalence of MDD (47.6%) was reported in a study conducted in the Awi Zone, in the Amhara region (Mulatu, Ejigu, et al. 2024), while the lowest prevalence (12.0%) was reported in Aleta Wondo Town, in SNNPR (Dafursa and Gebremedhin 2019) (Table 1). Details of the studies and their corresponding *p*‐values are presented in Table 2.

### 3.4 Meta‐Analysis

The pooled prevalence of MDD among children aged 6–23 months was estimated at 27% (95% CI: 21, 33). The findings showed substantial heterogeneity (*I* ^2^ = 97.58%, *p* < 0.001; *τ* ^2^ = 0.01; Q(12) = 414.67, *p* < 0.001), and the estimate should be interpreted cautiously, as shown in Figure 2a,b. Therefore, a random‐effects model was used.

**FIGURE 2:**
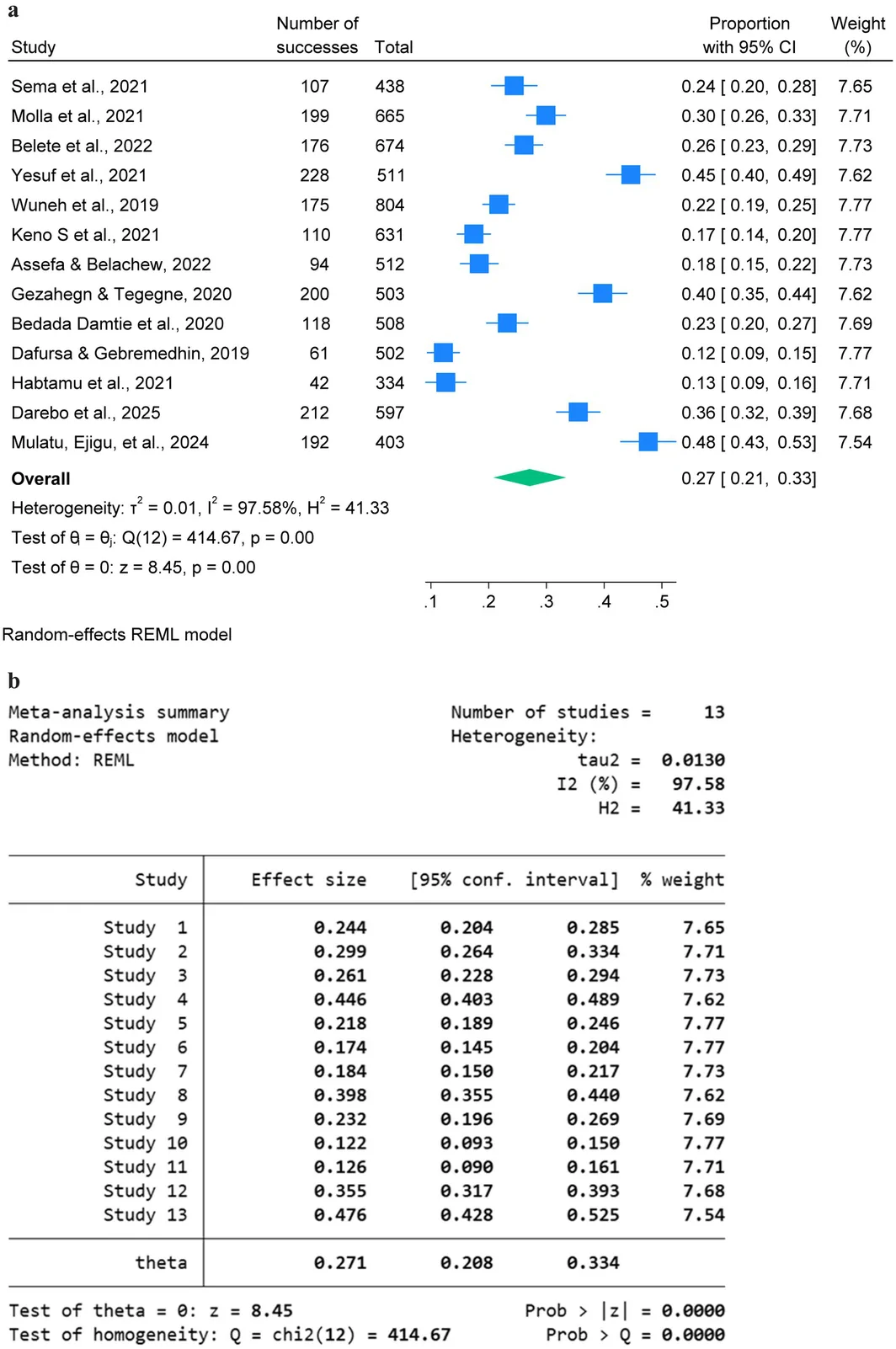
(a) Forest plot of the prevalence of minimal dietary diversity among children aged 6–23 months in Ethiopia (2018 to 2026). Pooled prevalence with 95% CIs was estimated using a random‐effects model (*I* ^2^ = 97.11%). (b) Summary of meta‐analysis for the prevalence of minimal dietary diversity among children aged 6–23 months in Ethiopia (2018 to 2026).

To assess the observed heterogeneity, subgroup analyses were conducted by publication year, region, sample size, and outcome definition; however, substantial heterogeneity persisted. Subgroup analysis by publication year (2018–2022 vs. 2022–2026) still showed high heterogeneity (*τ* ^2^ = 0.01, *I* ^2^ = 97.54%, Q(8) = 273.86, *p* < 0.001 vs. *τ* ^2^ = 0.02, *I* ^2^ = 97.66%, Q(3) = 108.78, *p* < 0.001) (Figure 3a). Similarly, subgroup analysis by sample size (large vs. small) indicated persistent heterogeneity (*τ* ^2^ = 0.01, *I* ^2^ = 96.90%, Q(6) = 160.50, *p* < 0.01 vs. *τ* ^2^ = 0.02, *I* ^2^ = 97.58%, Q(5) = 245.90, *p* < 0.001) (Figure 3b).

**FIGURE 3:**
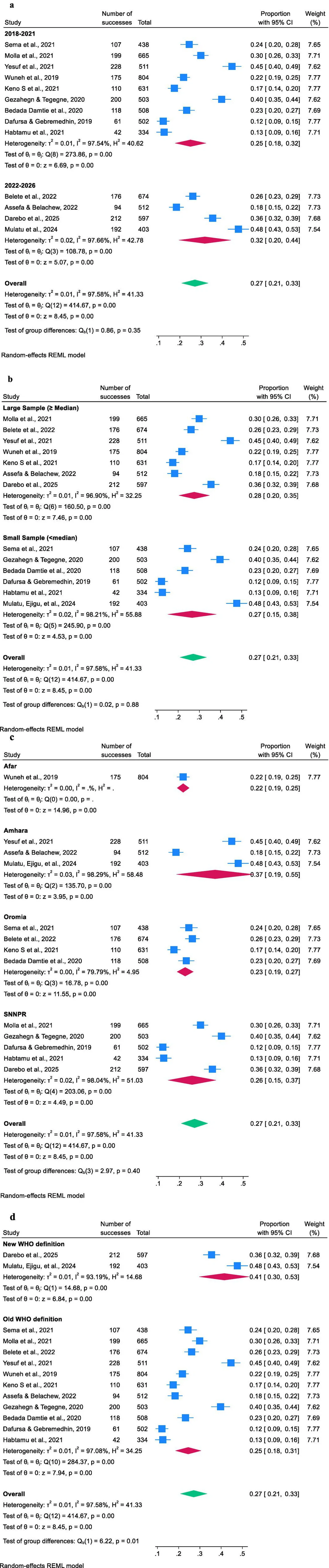
(a) Subgroup analysis by year of publication (2018–2021 vs. 2022–2026) for the prevalence of minimal dietary diversity among children aged 6–23 months in Ethiopia (2018 to 2026). Subgroup prevalence estimates (95% CIs) were generated using a random‐effects model (*I* ^2^ = 97.54 vs. 97.58, respectively). (b) Subgroup analysis by sample size (≥ median vs. < median) for the prevalence of minimal dietary diversity among children aged 6–23 months in Ethiopia (2018 to 2026). Subgroup prevalence estimates (95% CIs) were generated using a random‐effects model (*I* ^2^ = 96.9 vs. 98.21, respectively).(c) Subgroup analysis by region (Afar, Amhara, Oromia, and SNNPR) for the prevalence of minimal dietary diversity among children aged 6–23 months in Ethiopia (2018 to 2026). Subgroup prevalence estimates (95% CIs) were generated using a random‐effects model (*I* ^2^ = 0.01%, 98.28%, 79.79%, and 98.04%, respectively). (d) Subgroup analysis by outcome definition (new vs. old definition) for the prevalence of minimal dietary diversity among children aged 6–23 months in Ethiopia (2018 to 2026). Subgroup prevalence estimates (95% CIs) were generated using a random‐effects model (*I* ^2^ = 93.1% vs. 98.21%, respectively).

In addition, subgroup analysis by region (Afar, Amhara, Oromia, and SNNPR) showed substantial heterogeneity (*τ* ^2^ = 0.00, *I* ^2^ =. %, Q(0) = 0.00; Amhara, *τ* ^2^ = 0.03, *I* ^2^ = 98.29%, Q(2) = 135.7, *p* < 0.001; Oromia, *τ* ^2^ = 0.00, *I* ^2^ = 97.79%, Q(3) = 16.78, *p* < 0.001; SNNPR, *τ* ^2^ = 0.02, *I* ^2^ = 98.04%, Q(4) = 203.06, *p* < 0.001) (Figure 3c). However, subgroup analyses by geographic region and by sample size did not show statistically significant differences. Additionally, subgroup analysis using the WHO definition (old vs. new) showed substantial heterogeneity (*τ* ^2^ = 0.01, *I* ^2^ = 93.19%, Q(1) = 14.68, *p* < 0.001) versus (*τ* ^2^ = 0.01, *I* ^2^ = 97.08%, Q(19) = 284.37, *p* < 0.001) (Figure 3d). Therefore, cautious interpretation is warranted.

Publication bias among the studies included in the MDD prevalence assessment was assessed using funnel plots and Egger's test. The funnel plot appeared symmetrical, with evenly scattered points (Figure 4a), and Egger's test showed no evidence of small‐study effects or publication bias (*p* = 0.088) (Figure 4b), indicating the absence of publication bias.

**FIGURE 4:**
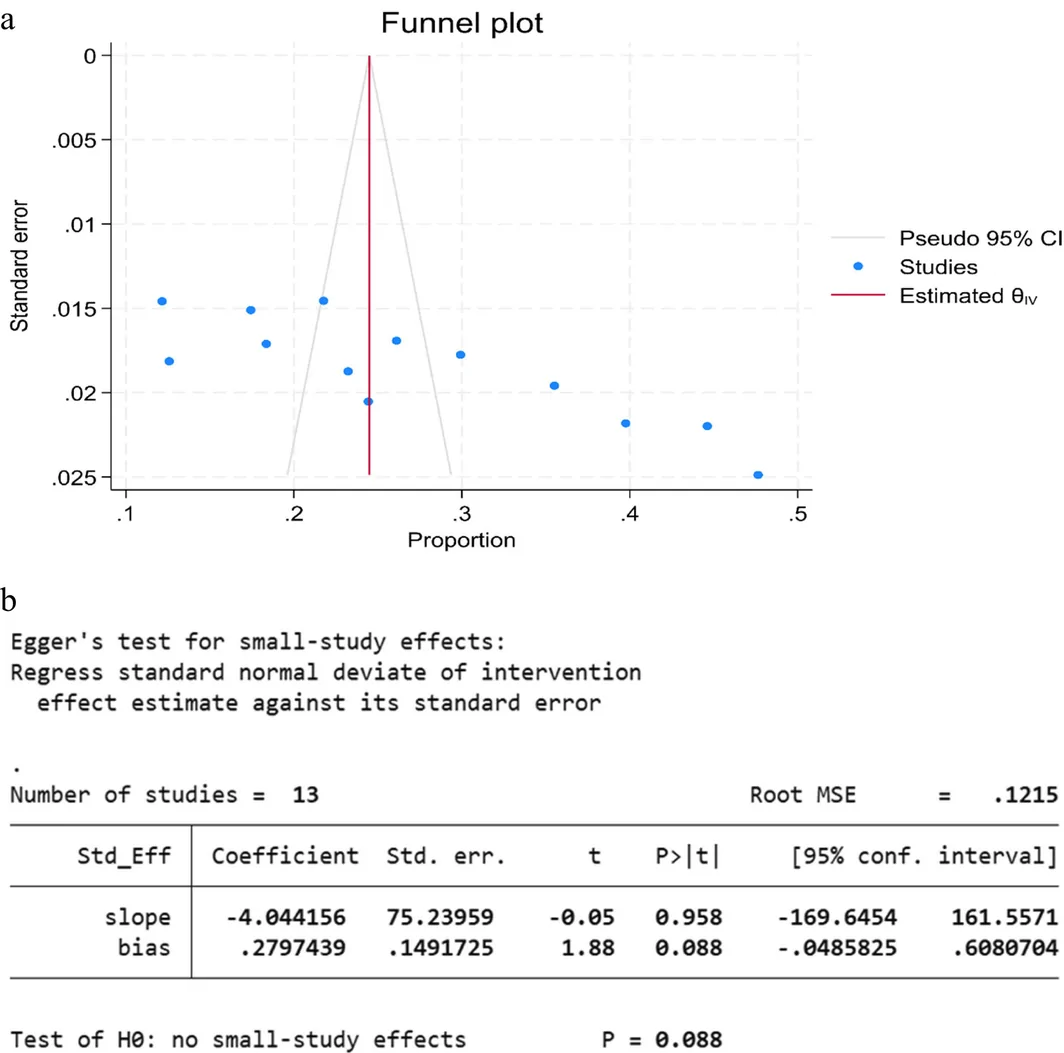
(a) Funnel plot for the prevalence of minimal dietary diversity among children aged 6–23 months in Ethiopia (2018 to 2026). (b) Egger's test for the prevalence of minimal dietary diversity among children aged 6–23 months in Ethiopia (2018 to 2026).

## 4 Discussion

### 4.1 Summary of Findings

The pooled prevalence of MDD among children aged 6–23 months in Ethiopia was 27% (95% CI: 21, 33). Due to substantial heterogeneity (*I* ^2^ = 97.58%, *p* < 0.001; *τ* ^2^ = 0.01; Q(12) = 414.67, *p* < 0.001), this finding requires cautious interpretation. Despite this limitation, it indicates that more than one in four children in this age group do not meet MDD recommendations for the required food groups in their daily diet. In a narrative synthesis of associated factors, several included studies reported child age, maternal education, ANC, PNC, household wealth index, and family size as statistically significant factors associated with MDD practice. These associations were reported in the original primary studies and were not derived from the meta‐analysis conducted in this review.

### 4.2 Heterogeneity

The meta‐analysis of MDD practice revealed substantial heterogeneity across the included studies. Subgroup analyses by year of publication, median sample size, WHO definition of MDD, and study region still revealed considerable heterogeneity, indicating that these subgroups did not fully account for the observed variation. The remaining heterogeneity may be attributed to variations in population characteristics and cultural, geographic, and socioeconomic contexts. Therefore, the pooled estimate should be interpreted with caution, particularly when comparing MDD prevalence and the reported associated factors across studies that use different methodological approaches and data sources. Only two of the 13 included studies applied the updated WHO definition of MDD. In addition, unmeasured factors, such as differences in feeding habits, seasonal food availability, and local health and nutrition programs, may have contributed to heterogeneity. Consequently, the pooled prevalence should be viewed as an average estimate rather than an indication of a consistent situation across different settings.

Based on the WHO MDD target of 40% for children aged 6–23 months by 2030 (World Health Organization 2024), our findings revealed a lower score. This low prevalence may be explained by socioeconomic and educational inequalities (Worku et al. 2025), limited knowledge of food diversification (Seyoum et al. 2024), and factors related to parental marital status and place of residence (Endawkie et al. 2025). Consistent with previous evidence, children from wealthier households and those whose mothers had higher educational attainment were more likely to achieve adequate dietary diversity (Paramashanti et al. 2022; Baffa et al. 2025). In Ethiopia, regional variations in dietary diversity are influenced by maternal education, household income, maternal health service, food availability, agricultural practices, market access, and cultural feeding behaviors (Alemu et al. 2022). In rural settings in Ethiopia, a multidimensional approach is needed, including enhancing market access, financial resources, and educational opportunities (Degfachew et al. 2025). This underscores the need for strengthening the local food system through strategies such as encouraging backyard gardening, hosting cooking demonstrations, expanding irrigation, improving communication access, and employing appropriate technologies (Asayehu et al. 2025), while also enhancing maternal education, financing, income, and maternal health services.

The pooled prevalence reported in this study is higher than that reported in various systematic reviews and meta‐analyses conducted in Ethiopia, which reported 26% (Mulatu, Mekonnen, et al. 2024), 23.25% (Temesgen et al. 2018) and 22% (Kassie et al. 2023), as well as several national surveys using HDS data, which reported 10.8% (Aemro et al. 2013), 7.8% (Disha et al. 2013), 4% (Ethiopian Demographic Health Survey (EDHS) 2011), 8.5% (Woldegebriel et al. 2020), 12.09% (Muche et al. 2022), and 12.5% (Sisay et al. 2022) MDD practice. Similarly, the observed prevalence is higher than findings from systematic reviews and meta‐analyses conducted using DHS data in Sub‐Saharan Africa, which reported 23.2% (Ba et al. 2022), 11% (Paulo et al. 2024), and in East Africa, which reported 10.47% (Raru et al. 2023). It is also higher than findings from individual studies conducted in Eastern and Southern Africa, including Uganda (15.2%) and South Africa (14.4%) (Kang et al. 2023). Furthermore, it exceeds findings from several Sub‐Saharan African countries, including Liberia (Ba et al. 2022), Burundi (16%) (Custodio et al. 2019), Burkina Faso (5.6%) (Aboagye et al. 2021) and Gambia (20.2%) (Aboagye et al. 2021).

However, the prevalence observed in this study is lower than that reported in several other countries and regions. Higher MDD prevalence has been reported in Rwanda (34.4%) (Ba et al. 2022), Indonesia (63.15%) (Sekartaji et al. 2021), Bangladesh (48.4%), Vietnam (83.2%) (Disha et al. 2013), Ghana (47.7%) (Issaka et al. 2015), Nepal (30.4%) (Khanal et al. 2013), Zambia (37%) (Disha et al. 2012), South Africa (43.9%) (Aboagye et al. 2021), and Sri Lanka (71%) (Senarath et al. 2012). In addition, evidence from Eastern and Southern Africa shows higher prevalence in countries such as Zimbabwe (31.1%), Kenya (40.3%), Malawi (26.5%), and Rwanda (30.5%) (Kang et al. 2023). These variations in dietary diversity across countries may be attributed to multiple contextual factors. Africa is characterized by diverse food systems, cultural practices, and dietary habits that vary across settings (The African Nutrishinist 2025). Differences in nutrition education (Antwi and Janet 2020), religious and cultural practices (Onward Living HQ 2025), and climate change, including rising temperatures and increased rainfall variability, also have a profound impact on dietary diversity (Niles et al. 2021), as well as broader determinants, including historical influences, globalization, socioeconomic conditions, and social networks, play an important role in shaping dietary behaviors (Jayasinghe et al. 2025). Methodological and data‐related factors may also explain these differences. Most of the referenced studies used DHS data, which are secondary, whereas some reviews included a mix of community‐ and institution‐based studies. Sampling techniques, sample sizes, geographic coverage, and data collection tools may also influence the reported prevalence. In addition, variations in outcome definitions, study populations, settings, and data collection periods may contribute to the observed differences. These factors could further influence the reported estimate. Differences in national IYCF programs and policies also contribute to variations in dietary diversity. Countries with stronger nutrition programs, higher maternal education, and more comprehensive health services tend to report better outcomes. Therefore, differences observed across countries and regions should be considered to result from nutritional status. However, they should also be interpreted within the broader contexts of policies, socioeconomic factors, and methodologies.

Lower MDD practice and its associated factors can be addressed through targeted policy and programmatic interventions. These include improving complementary feeding practices and nutrition counseling (Darnton‐Hill 2017), strengthening nutrition education (Rees et al. 2022), implementing nutrition‐sensitive interventions (De Hoop et al. 2025), and expanding social protection and food access programs (Escher et al. 2024). Additional strategies include improving parental knowledge and feeding practices (Chan et al. 2025), strengthening food, health, and social protection systems (United Nations Children's Fund (UNICEF) 2020), and promoting backyard gardening, cooking demonstrations, access to irrigation, improved communication access, and the use of suitable technology (Asayehu et al. 2025). Efforts to improve dietary diversity should also address broader structural factors, including the food system, health service infrastructure, market access, affordability, diverse farming systems (United Nations Children's Fund (UNICEF) 2020; Hailu et al. 2026), and targeted nutrition interventions (Hailu et al. 2026). In Ethiopia, progress toward food and nutrition security is constrained by environmental, nutritional, and technological factors, as well as by emerging outbreaks and political instability. However, action to address challenges and the translation of available evidence into practice are required (Brhanu et al. 2023). Given the multifactorial nature of inadequate dietary diversity, a multisectoral approach is a promising strategy for achieving sustainable improvements in child feeding practices (Ayele et al. 2026). Despite some progress, the persistently low prevalence of MDD underscores the context‐specific need for interventions to improve child nutrition practices.

### 4.3 Strengths and Limitations

This study provides updated evidence, incorporates community‐based studies, focuses on the critical age for malnutrition, includes studies from different regions of Ethiopia, and applies a systematic review and meta‐analysis approach. All included studies were adjusted for confounders. It also includes the most recent research conducted between 2018 and 2026. However, the study has some limitations. It includes only cross‐sectional studies, which limits the ability to establish a temporal relationship between exposure and outcome. There is heterogeneity across studies, and two of the 13 studies used the new MDD definition. The study synthesizes factors rather than performing a pooled analysis. Additionally, because all included studies are cross‐sectional, the outcome variables may have been influenced by confounding factors. Differences in study settings, survey periods, and data sources may have influenced the reported estimates.

## 5 Conclusions

Based on the WHO target for 2030, our findings indicate that the prevalence of MDD among children aged 6–23 months in Ethiopia remains low (27%). However, substantial heterogeneity across studies limits the precision and generalizability of the pooled estimates. Among the included studies, child age, maternal education, ANC, PNC, and household wealth index were the factors most frequently reported to be significantly associated with MDD in multivariable‐adjusted analyses. These findings highlight the need for targeted interventions to improve feeding practices and support the achievement of SDG 2.2. Strengthening maternal education and improving maternal healthcare utilization may contribute to improvements in child dietary diversity in Ethiopia.

Because considerable heterogeneity was observed across studies, which was not explained by subgroup analysis, both the pooled prevalence estimate and the narratively synthesized evidence on factors reported as significantly associated with MDD in the included studies should be interpreted with caution.

## Author Contributions

**Melkamu Senbeta Jimma:** conceptualization, methodology, software, data curation, formal analysis, investigation, supervision, visualization, writing – original draft, writing – review and editing, project administration, validation, resources. **Dawit Getachew Assefa:** conceptualization, methodology, software, investigation, formal analysis, writing – review and editing, writing – original draft, validation. **Muluwas Amentie Zelka:** writing – review and editing, supervision, visualization, conceptualization. **Alemayehu Assefa Amaya:** supervision, writing – review and editing, visualization, conceptualization. **Mulualem Birhanu Begawo:** writing – review and editing, visualization, supervision, conceptualization. **Ermias Sisay Chanie:** methodology, conceptualization, validation, writing – review and editing, data curation, supervision. **Fisha Alebel Gebreeyesus:** methodology, supervision, validation, writing – review and editing, data curation, investigation, resources. **David Lim:** validation, methodology, visualization, writing – review and editing, supervision, resources, project administration, conceptualization, writing – original draft, data curation, funding acquisition.

## Funding

The authors have nothing to report.

## Ethics Statement

Ethical review and approval were waived for this study because it used publicly available data and did not involve human participants.

## Conflicts of Interest

The authors declare no conflicts of interest.

## Supporting information


**File S1:** Supporting information.

## Data Availability

The data that support the findings of this study are available from the corresponding author upon reasonable request.
