# Supplementary material for: Minimum Dietary Diversity and Associated Factors Among 6–23 Months Children in Ethiopia: A Systematic Review and Meta‐Analysis
Source: Food Sci Nutr. 2026 Oct 13;14(10):e72481. doi: 10.1002/fsn3.72481 (PMC13661749; doi:10.1002/fsn3.72481)
Supplement: Supplementary file 1 [file FSN3-14-e72481-s001.docx]

**Search strategy**

**PubMed:**

*((“Diet Diversity” [Mesh] OR "minimum dietary diversity"[Title/Abstract] OR "dietary diversity score"[Title/Abstract]) AND ("complementary feeding"[Title/Abstract] OR "infant feeding practices"[Title/Abstract]) AND ("associated factors"[Title/Abstract] OR "determinants"[Title/Abstract]) AND ("children aged 6-23 months"[Title/Abstract] OR "infants"[Title/Abstract]) AND ("Ethiopia"[Title/Abstract]))* Filters: English; Publication date: 01 April 2018 to 25 April 2026

**Embase (Ovid):**

*(‘diet diversity’/exp OR 'minimum dietary diversity':ti,ab OR 'dietary diversity score':ti,ab) AND ('complementary feeding' OR 'infant feeding practices*':ti,ab*) AND ('associated factors' OR 'determinants') AND ('children aged 6-23 months' OR 'infants') AND ('Ethiopia').* 'infant'/exp OR 'children aged 6-23 months':ti,ab) AND ('ethiopia'/exp OR ethiopia:ti,ab) AND ('risk factor'/exp OR determinant*:ti,ab OR 'associated factor*':ti,ab) Limits: English; 2018-2026

**Web of Science**: TS=(("minimum dietary diversity" OR "dietary diversity score") AND ("complementary feeding" OR "infant feeding practices") AND ("associated factors" OR determinants) AND ("children aged 6-23 months" OR infants) AND (Ethiopia)) Refined by: English language; Refined by: English language; Publication years 2018-2026

**Cochrane Library:** ("minimum dietary diversity" OR "dietary diversity score") AND ("complementary feeding" OR "infant feeding practices") AND ("associated factors" OR determinants) AND ("children aged 6-23 months" OR infants) AND (Ethiopia) Limits: English; 2018-2026

**Google Scholar:**

"Minimum dietary diversity" AND Ethiopia AND ("associated factors" OR determinants) AND ("children aged 6-23 months" OR infants) Filters: English; Custom date range: 2018-2026

**HINARI:**

("minimum dietary diversity" OR "dietary diversity score") AND ("complementary feeding" OR "infant feeding practices") AND ("associated factors" OR determinants) AND ("children aged 6-23 months" OR infants) AND (Ethiopia) Language: English; Limits: English; 2018-2026
